# Integrated multi-omics analysis reveals the mechanisms of naringin in ameliorating high-fat diet-induced metabolic dysfunction-associated steatotic liver disease

**DOI:** 10.3389/fnut.2025.1694191

**Published:** 2025-10-21

**Authors:** Wenping Sun, Na Xue, Qiang Zhang

**Affiliations:** ^1^Surgery of Hepatobiliary and Pancreatic Cancer, Gansu Provincial Cancer Hospital, Lanzhou, Gansu, China; ^2^School of Basic Medicine, Gansu University of Chinese Medicine, Lanzhou, Gansu, China; ^3^Urology Surgery, Gansu Provincial Cancer Hospital, Lanzhou, Gansu, China

**Keywords:** naringin, MASLD, lipid metabolism, gut microbiota, network pharmacology

## Abstract

**Introduction:**

Naringin (Nar), the predominant flavonoid in citrus fruits, shows therapeutic potential against metabolic dysfunction-associated steatotic liver disease (MASLD). However, its underlying mechanisms remain largely elusive.

**Methods:**

In this study, we investigated the efficacy and underlying mechanisms of Nar in a mouse model of high-fat diet (HFD)-induced MASLD using integrated analyses of network pharmacology, molecular docking, hepatic lipidomics, and gut microbiota.

**Results:**

Treatment with Nar markedly ameliorated MASLD phenotypes, as evidenced by reduced body and liver weights, lower hepatic triglycerides (TGs), and improved serum alanine aminotransferase (ALT) and aspartate aminotransferase (AST) levels. Network pharmacology analysis revealed that Nar targets associated with MASLD are primarily enriched in proteins such as SRC, AKT1, STAT3, FOS, ESR1, and NFKB1, which exert their effects through the PI3K-AKT signaling pathway. Molecular docking simulations further elucidated the interaction mechanisms. Lipidomic analysis revealed that Nar restored hepatic lipid homeostasis, significantly decreasing levels of TGs and diglycerides (DGs), with 20 differentially abundant lipid species identified as potential biomarkers. Additionally, Nar profoundly altered the gut microbial community, promoting the enrichment of beneficial genera including *Oscillibacter*, *Allisonella*, and *Flavonifractor*.

**Discussion:**

Our findings indicate that Nar prevents MASLD by harmonizing hepatic lipid metabolism and modulating the gut microbiome, providing a multifaceted mechanistic insight into its therapeutic potential.

## 1 Introduction

Metabolic dysfunction-associated steatotic liver disease (MASLD) has emerged as a global chronic liver disorder, affecting approximately 25–30% of adults worldwide, with its prevalence rising alongside that of metabolic syndrome and obesity (1, 2). Clinically defined by excessive hepatic lipid accumulation independent of alcohol use, MASLD represents a progressive condition spanning simple steatosis, non-alcoholic steatohepatitis, cirrhosis, and hepatocellular carcinoma (2–4). Its etiology is deeply interwoven with insulin resistance, dysregulated lipid metabolism, and gut microbiota dysbiosis, thereby functioning as a hepatic manifestation of metabolic syndrome (5–7). Despite its growing public health burden, effective pharmacotherapy remains elusive, leaving lifestyle modifications—often inadequate—as the primary management strategy (8–10).

The therapeutic bottleneck in MASLD stems largely from an incomplete dissection of its multifactorial pathogenesis (11). While lipidomic disruptions, particularly elevated triglycerides (TGs) and cholesterol esters, are well-characterized drivers of hepatic steatosis, accumulating evidence highlights the gut microbiota as a master regulator of lipid homeostasis and inflammation. Moreover, intestinal flora is closely linked to alterations in host liver metabolites. Recent studies suggest that bile acids, short-chain fatty acids, trimethylamine N-oxide, and tryptophan metabolites are critical in driving hepatic inflammation and steatosis, thereby influencing MASLD progression. Therefore, this positions the gut–liver axis as a promising therapeutic target for MASLD intervention (12, 13).

Natural flavonoids, particularly naringin (Nar)—an abundant bioactive constituent of citrus fruits—have demonstrated considerable potential in alleviating metabolic dysfunction (14). Preclinical investigations revealed Nar’s capacity to reduce hepatic TG levels, suppress oxidative stress, and modulate pro-inflammatory signaling cascades (e.g., NF-κB pathway) (15). To systematically dissect the complex interplay between host metabolism and microbial communities, advanced omics technologies offer indispensable tools. Lipidomics, in particular, enables high-resolution mapping of hepatic lipid signatures, providing insights into diagnostic biomarkers and mechanistic pathways (16–18). Although widely used to evaluate traditional Chinese medicine (TCM) formulations, conventional lipidomics faces limitations in deconvoluting specific bioactive compounds and their lipidomic protein interactions (18). Integration with network pharmacology and lipidomic modeling addresses this gap, facilitating precise identification of active constituents, prediction of molecular targets, and elucidation of synergistic mechanisms (19). Concurrently, the gut microbiota emerges as a central node in metabolic regulation, with dietary interventions shown to mitigate MASLD severity via microbiota-mediated alterations in host lipid metabolism.

Using a combination of network pharmacology and molecular docking, we aimed to evaluate the potential of Nar-derived metabolites involved in lipid metabolism modulation. To validate this hypothesis experimentally, high-fat diet (HFD)-fed mice were treated with Nar, followed by integrated omics analyses to characterize hepatic metabolic shifts and gut microbial community dynamics. Our findings provide novel insights into the multi-target actions of Nar in MASLD amelioration, offering a foundation for developing microbiota-directed therapeutic strategies.

## 2 Materials and methods

### 2.1 Chemicals and reagents

Naringin was purchased from Chengdu Munster Biotechnology Co., Ltd. (Chengdu, China). Liquid chromatography–mass spectrometry (LC–MS) grade acetonitrile, methanol, isopropanol, and formic acid were supplied by Fisher Scientific (Fisher Scientific, California, United States), and distilled water was purchased from Watsons (Guangzhou Watsons Food & Beverage Co., Ltd., China).

### 2.2 Animal experiments

A total of 18 male C57BL/6 mice (6–7 weeks old) were obtained from the Chinese Academy of Medical Sciences (Beijing, China). These mice were maintained in a specific pathogen-free laboratory animal center under controlled conditions (temperature 22 ± 2 °C, humidity 55 ± 5%, and a 12-h light/dark cycle) with free access to food and water. Following 1 week of adaptation, the mice were randomly divided into three groups (*n* = 6): a normal chow diet group (Chow, 12% fat), a high-fat diet group (HFD, 60% fat), and a high-fat diet with Nar group (HFD supplemented with 0.07% Nar). The 0.07% NAR dose was selected based on prior research (20). All experimental procedures adhered to the Guide for the Care and Use of Laboratory Animals (National Institutes of Health) and were approved by the Animal Ethics Committee of Gansu University of Traditional Chinese Medicine (SYXK(Gansu)2024-0005).

### 2.3 Sample collection and preparation

After a 12-week experimental intervention, mice were fasted for 12 h and subsequently euthanized under pentobarbital sodium anesthesia. Blood samples, liver tissues, and epididymal adipose tissues were collected for further analysis. Serum was isolated through centrifugation at 3,000 rpm for 15 min (4 °C) and preserved at −80 °C for subsequent biochemical analyses.

### 2.4 Histopathological evaluation

Histopathological analysis was performed as previously described (20). In summary, livers fixed in 4% paraformaldehyde were processed into paraffin sections with a thickness of 5 μm. These sections were stained with hematoxylin and eosin (H&E) for histological examination. The stained sections were then mounted with neutral resin, and images were captured under a microscope at 400 × magnification.

### 2.5 Biochemical analysis

The serum samples were retrieved from the −80 °C freezer and allowed to thaw at 4 °C. The levels of TGs in the liver tissue, as well as serum aspartate transaminase (AST) and alanine aminotransferase (ALT), were measured using activity assay kits (Jiancheng Biotech, Nanjing, China) following the manufacturer’s instructions.

### 2.6 Hepatic lipidomic analysis

For sample preparation, 25 mg of liver tissue from each group (*n* = 6) was used. The liver tissue was powdered and mixed with 300 μL of phosphate-buffered saline (PBS) by vortexing. Subsequently, 429 μL of methyl tert-butyl ether, 342 μL of methanol, and 429 μL of water were added to the mixture, which was vortexed for 1 min and then centrifuged at 3,000 rpm for 15 min at 4 °C. The supernatant (300 μL) was collected and evaporated at −20 °C. After drying, 100 μL of isopropanol was added to the dried mixture, vortexed on ice for 3 min, and centrifuged at 13,000 rpm for 15 min at 4 °C. The final supernatant was transferred to a glass vial for UPLC/MS analysis. The QC samples were prepared by mixing equal volumes (20 μL each) of all the samples.

Lipidomic analysis was performed using an ExionLC Infinity series ultra-high performance liquid chromatography (UPLC) system (AB Sciex) equipped with a BEH C18 column (Waters, 2.1 × 100 mm, 1.7 μm) maintained at a column temperature of 45 °C. The mobile phase consisted of two solvents: Solvent A was water/acetonitrile containing 10 mM ammonium formate (40:60, v/v), and Solvent B was a mixture of 10% acetonitrile/isopropanol (10:90, v/v) with 50 mL of 10 mM ammonium formate per 1,000 mL. The gradient elution was as follows: 0–12 min, 2–45% B; 12–20 min, 45–65% B; 20–25 min, 65–99% B; 25–26 min, 99–2% B; and 26–30 min, 2% B. The flow rate was set at 0.3 mL/min, and the injection volume was 3 μL (for both positive and negative ion modes). MS/MS spectra were acquired using a TripleTOF 5,600 mass spectrometer. Data analysis was conducted according to previously described methods (18). Raw UPLC-Q-TOF/MS data were processed using PeakView software (SCIEX) for peak picking, alignment, and integration. To ensure the quality of the data, endogenous metabolites were filtered based on a relative standard deviation (RSD) of < 30% in the quality control (QC) samples, thereby eliminating variations that could compromise reliability. The data were subsequently normalized to the total peak area to correct for systematic variations in signal intensity across samples (clearer explanation of normalization). Multivariate statistical analysis was then performed using SIMCA (version 14.1), where an orthogonal partial least squares-discriminant analysis (OPLS-DA) model was constructed to assess group separation and calculate the variable importance in projection (VIP) scores. Differential metabolites were identified based on a VIP value > 1.0 and a Student’s *t*-test *p*-value < 0.05, ensuring the selection of statistically significant metabolites. Metabolite identification was conducted by comparing accurate mass and MS/MS fragmentation patterns with entries from the LIPID MAPS database and other public databases. Finally, pathway enrichment analysis was performed on these significantly altered metabolites using MetaboAnalyst 5.0 to identify potential biological pathways.

### 2.7 Analysis of network pharmacology analysis and molecular docking

Potential targets of Nar were retrieved from multiple databases, including SwissTargetPrediction, TCMSP, PharmMapper, and the Comparative Toxicogenomics Database. NASH-related targets were obtained from GeneCards, the Therapeutic Target Database, and OMIM. All retrieved targets were standardized using the UniProt database. Venn diagram analysis was performed to identify intersecting targets between MASLD and Nar. A protein–protein interaction (PPI) network of the overlapping targets was constructed using the STRING database and visualized using Cytoscape 3.9.1. Kyoto Encyclopedia of Genes and Genomes (KEGG) pathway enrichment analysis was subsequently conducted on the intersecting targets. Molecular docking was carried out using AutoDock Tools 1.5.7 to predict the binding interactions between NAR and key targets, and the results were visualized using PyMOL software.

### 2.8 Analysis of gut microbiota composition

Full-length 16S rRNA gene amplicon sequencing of the fecal gut microbiome was performed by Novogene (Beijing, China). The V1–V9 hypervariable regions of the bacterial 16S rRNA gene were amplified using the universal primers 27F (5′–AGAGTTTGATCCTGGCTCAG–3′) and 1492R (5′–GNTACCTTGTTACGACTT–3′). Equimolar pooled PCR products were purified and subjected to sequencing on the PacBio platform. Sample demultiplexing was performed based on barcode sequences using Lima. After removing primers using Cutadapt, sequences underwent Small Subunit Ribosomal RNA (SSR) filtering. Quality-filtered reads were clustered into operational taxonomic units (OTUs) at 97% sequence identity using UPARSE. Representative sequences from each OTU were taxonomically annotated against the Silva SSU rRNA database with Mothur. The relative abundance of OTUs across samples was calculated for subsequent analysis.

### 2.9 Data processing and multivariate analysis

All experimental data are presented as mean ± standard deviation (SD) of the means. The sample means were analyzed with a one-way ANOVA using SPSSV20 software (IBM, Armonk, NY, United States). GraphPad Prism 8 (San Diego, CA, United States) was used for Figure preparation. Differences between the groups with a *p*-value of <0.05 were considered significant.

## 3 Results

### 3.1 Predicting MASLD alleviation by NAR via network pharmacology and molecular docking

**Figure 1:**
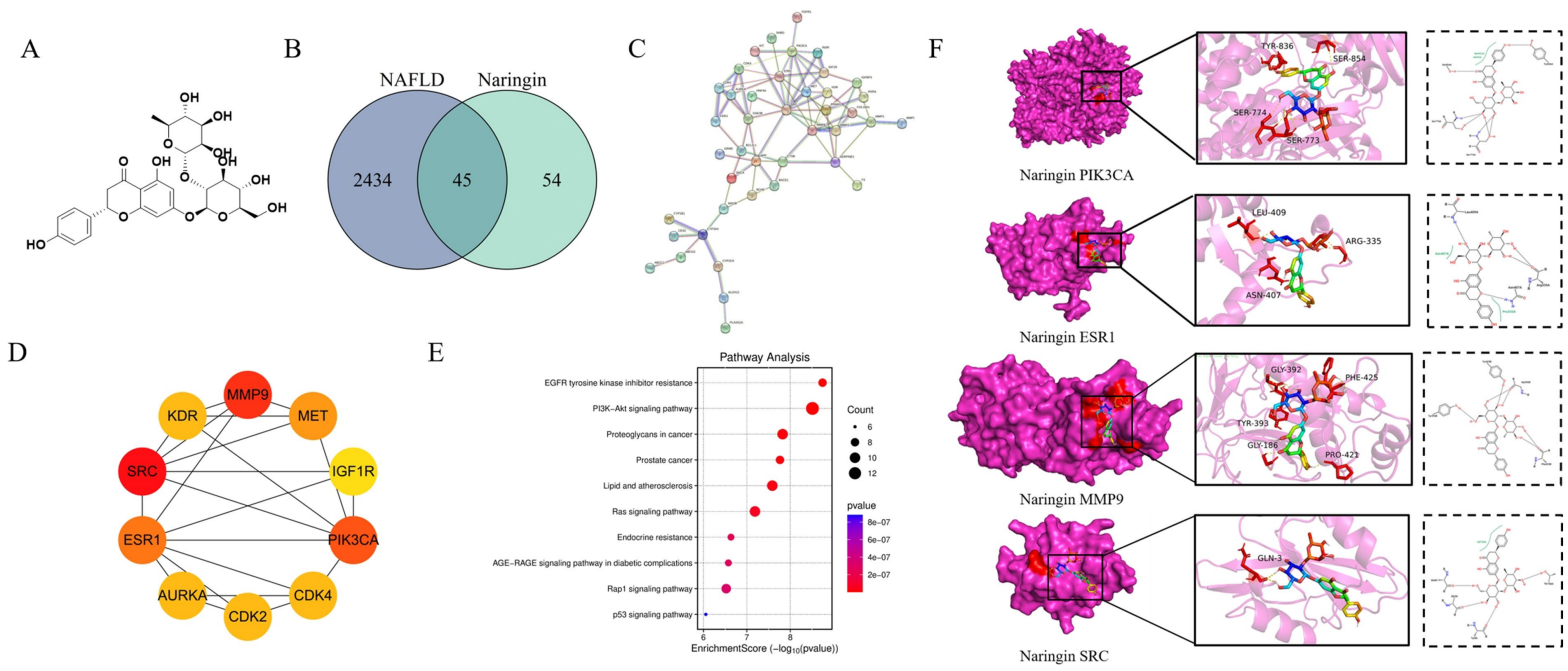
Network pharmacology analysis of the mechanism of Nar in MASLD. **(A)** Chemical structure of Nar. **(B)** Venn diagram illustrating the overlap between MASLD-related genes and Nar-associated genes. **(C)** Protein–protein interaction (PPI) network constructed based on the identified targets, highlighting key interactions. **(D)** Functional enrichment analysis revealing biological processes, cellular components, and molecular functions related to MASLD. **(E)** Pathway analysis identifying significant signaling pathways associated with MASLD. **(F)** Molecular docking results showing the binding interactions between Nar and its target proteins.

To elucidate the molecular mechanisms underlying NAR regulation of MASLD, a network pharmacology approach was used. By overlapping 99 NAR-related targets, 2,479 MASLD-related targets, and 45 potential common targets, they were identified as being involved (Figure 1B). Using STRING and Cytoscape, a PPI network was constructed, and topological analysis identified six core targets: AKT1, SRC, STAT3, FOS, ESR1, and NFKB1 (Figures 1C,D). These targets laid the foundation for elucidating the molecular mechanisms of nar’s multi-target regulation of MASLD. KEGG enrichment analyses using the DAVID database revealed that these targets are primarily involved in the P13K–Akt signaling pathway and lipid and atherosclerosis, underscoring their relevance to MASLD pathogenesis (Figure 1E). Molecular docking validation revealed strong binding affinity between Nar and the four core targets (AKT1, SRC, STAT3, FOS, ESR1, and NFKB1). Molecular docking of MASLD targets demonstrated that key targets in the PIK3CA, ESR1, MMP9, and SRC protein receptors exhibited spontaneous binding. Notably, the binding energies of PIK3CA, ESR1, MMP9, and SRC were all <−5 kcal/mol, indicating strong affinity for these targets (Figure 1F).

### 3.2 The protective effects of Nar on the MASLD mice induced by high-fat diet

**Figure 2:**
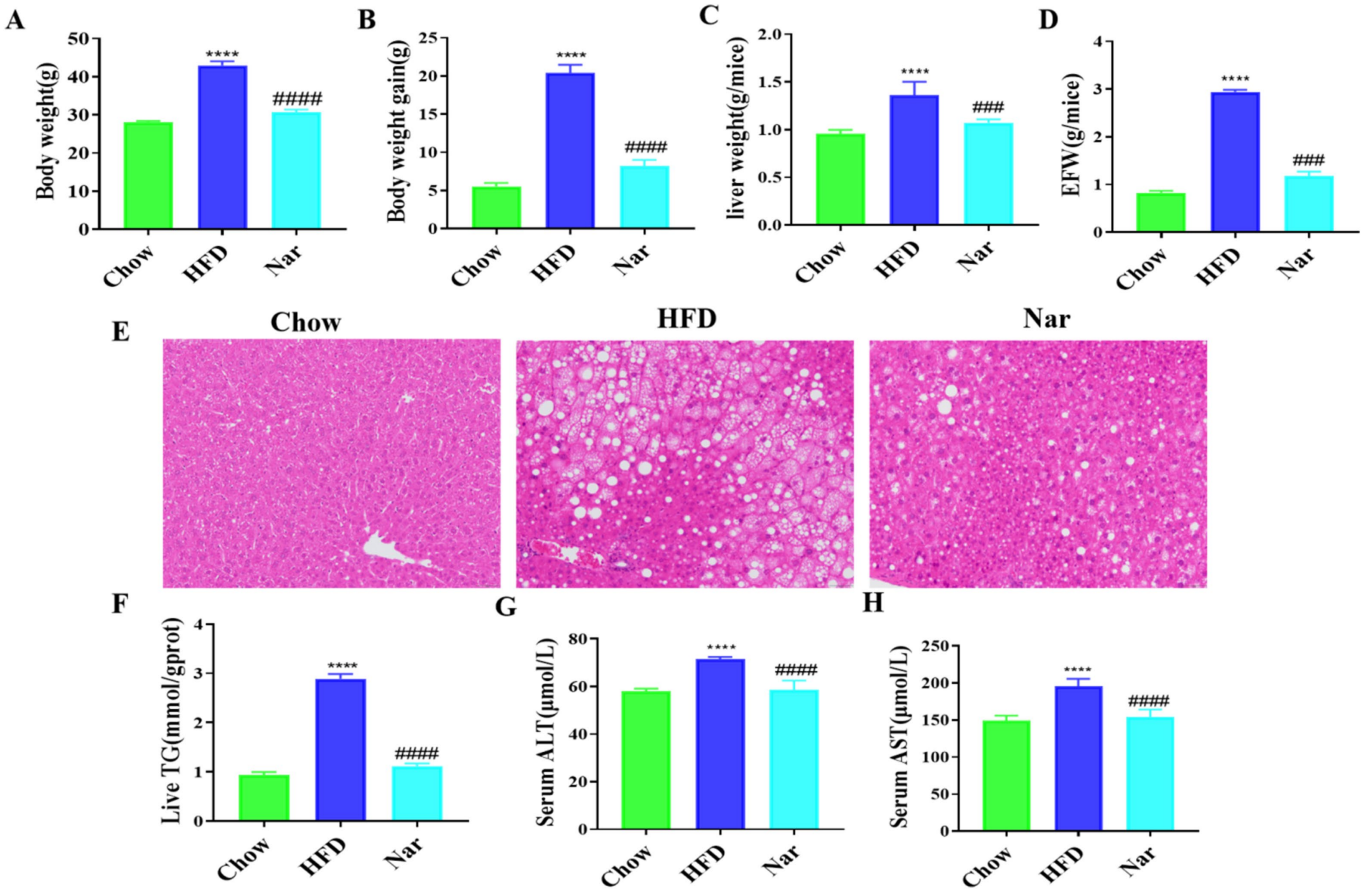
Nar attenuates metabolic dysregulation in high-fat diet-induced MASLD mice. **(A)** Body weight of each group of mice. **(B)** Body weight again. **(C)** Absolute liver weight. **(D)** Epididymal fat tissue weight (EFW). **(E)** Hepatic pathology sections H&E (scale bar, 200 μm). **(F)** Hepatic TG levels. **(G)** Serum levels of ALT. **(H)** Serum levels of AST; *n* = 6 each group, ****p < 0.0001, versus Chow group; ^####^*p* < 0.0001 versus HFD group.

The growth parameters are shown in Figures 2A–D. The HFD group exhibited significant increases in body weight, body weight gain, liver weight, and epididymal fat tissue (EFT) weight compared to the Chow group (*p* < 0.05 for all). After 12 weeks of intervention, the Nar group displayed significantly lower body weight, body weight gain, and liver weight than the HFD group (*p* < 0.05 for all). Additionally, the Nar group had a significantly lower EFT weight than the HFD group (*p* < 0.05).

Histological analysis, as shown in Figure 2E, revealed that liver tissue from the Chow group exhibited neatly arranged hepatocytes with minimal lipid accumulation, whereas liver tissue from the HFD group showed diffuse hepatic fatty infiltration with numerous fat vacuoles. Notably, hepatic steatosis in the HFD mice was markedly reduced following Nar intervention. Furthermore, hepatic triglyceride (TG) levels, which were elevated in the HFD group, were significantly reduced after Nar treatment (Figure 2F). The serum levels of ALT and AST, well-known biomarkers of hepatic injury, are presented in Figures 2G,H. The HFD group exhibited significantly elevated serum ALT and AST levels compared to the Chow group (*p* < 0.05). However, Nar supplementation significantly reduced serum ALT and AST activities in the HFD group. Collectively, these results indicate that Nar intervention effectively ameliorates hepatic lipid deposition and dysfunction induced by the HFD.

### 3.3 Influence of lipidomic profiles in the liver of MASLD mice by high-fat diet

**Figure 3:**
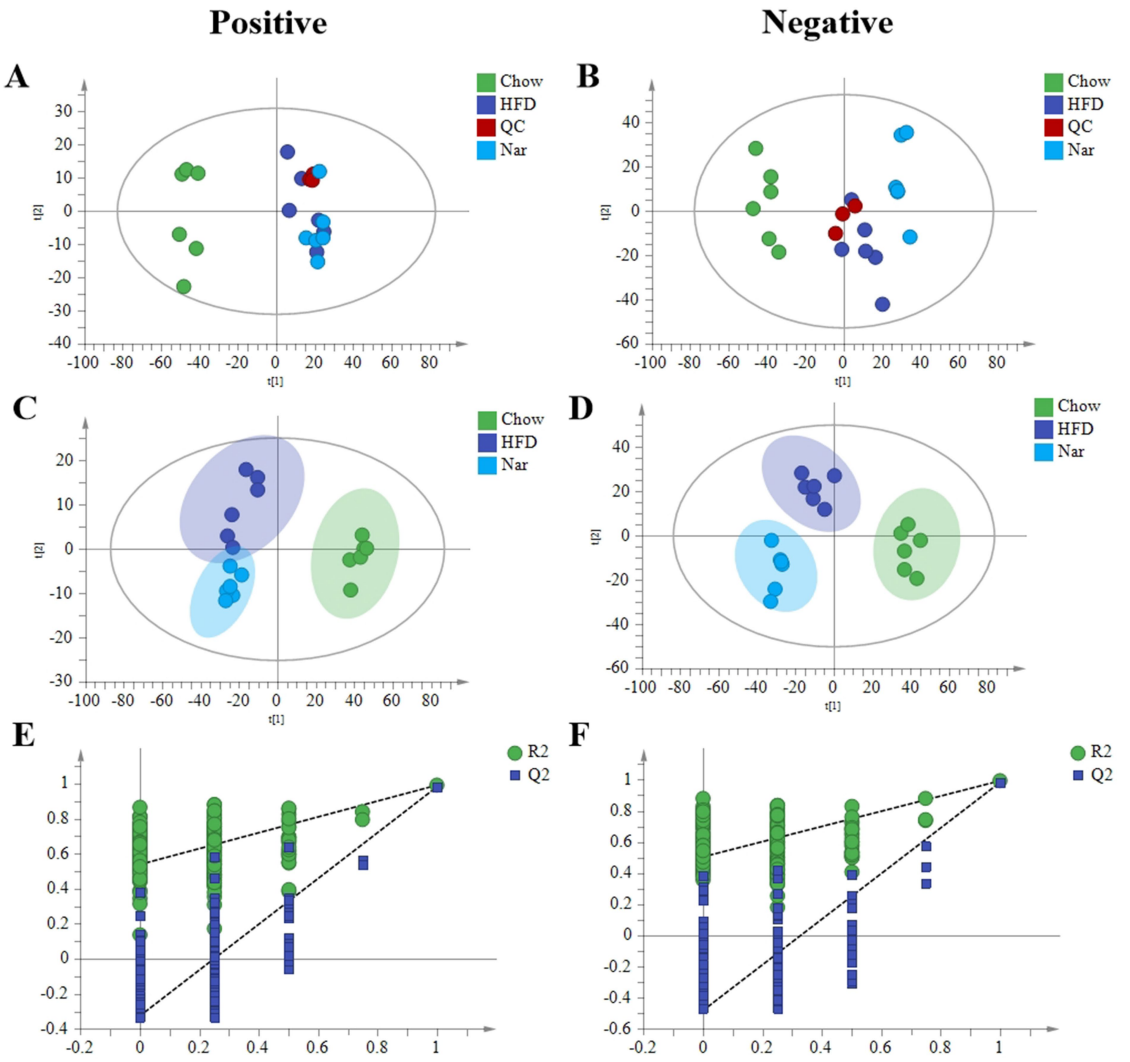
Lipidomic analysis by HPLC-MS/MS. **(A)** PCA score plots in the positive mode. **(B)** PCA score plots in the negative mode. **(C)** PLS-DA score plots in the positive mode. **(D)** PLS-DA score plots in the negative mode. **(E)** 200 permutations of the liver lipid PLS-DA model in the positive mode; **(F)** 200 permutations of the liver lipid PLS-DA model in the negative mode.

Increasing evidence suggests that the pathophysiology of MASLD is primarily characterized by an imbalance between lipid acquisition and lipid disposal (21). To further elucidate the regulatory mechanisms of Nar, we performed a lipidomic analysis on liver tissue. Principal Component Analysis (PCA) analysis revealed clear separation of the Chow, HFD, and Nar groups in both positive and negative ion modes, indicating significant differences in hepatic metabolic profiles among the groups. The QC samples were well-clustered, demonstrating the stability and reliability of the analysis system (Figures 3A,B). To achieve better group separation, we applied a supervised PLS-DA model to analyze the liver tissue samples. The PLS-DA results showed distinct separation between the Chow and HFD groups, with the Nar group closer to the Chow group (Figures 3C,D), suggesting that Nar partially reversed the lipid metabolism disorder induced by the HFD. The model was validated by 200 random permutation tests, yielding R^2^Y and Q^2^ values of 0.987 and 0.947 in the positive ion mode and 0.96 and 0.815 in the negative ion mode, respectively. These results confirm that the model is not overfitted and possesses strong predictive capability and reliability (Figures 3E,F). Collectively, these findings provide evidence that Nar supplementation can reverse the lipid metabolism disorder caused by HFD.

**Figure 4:**
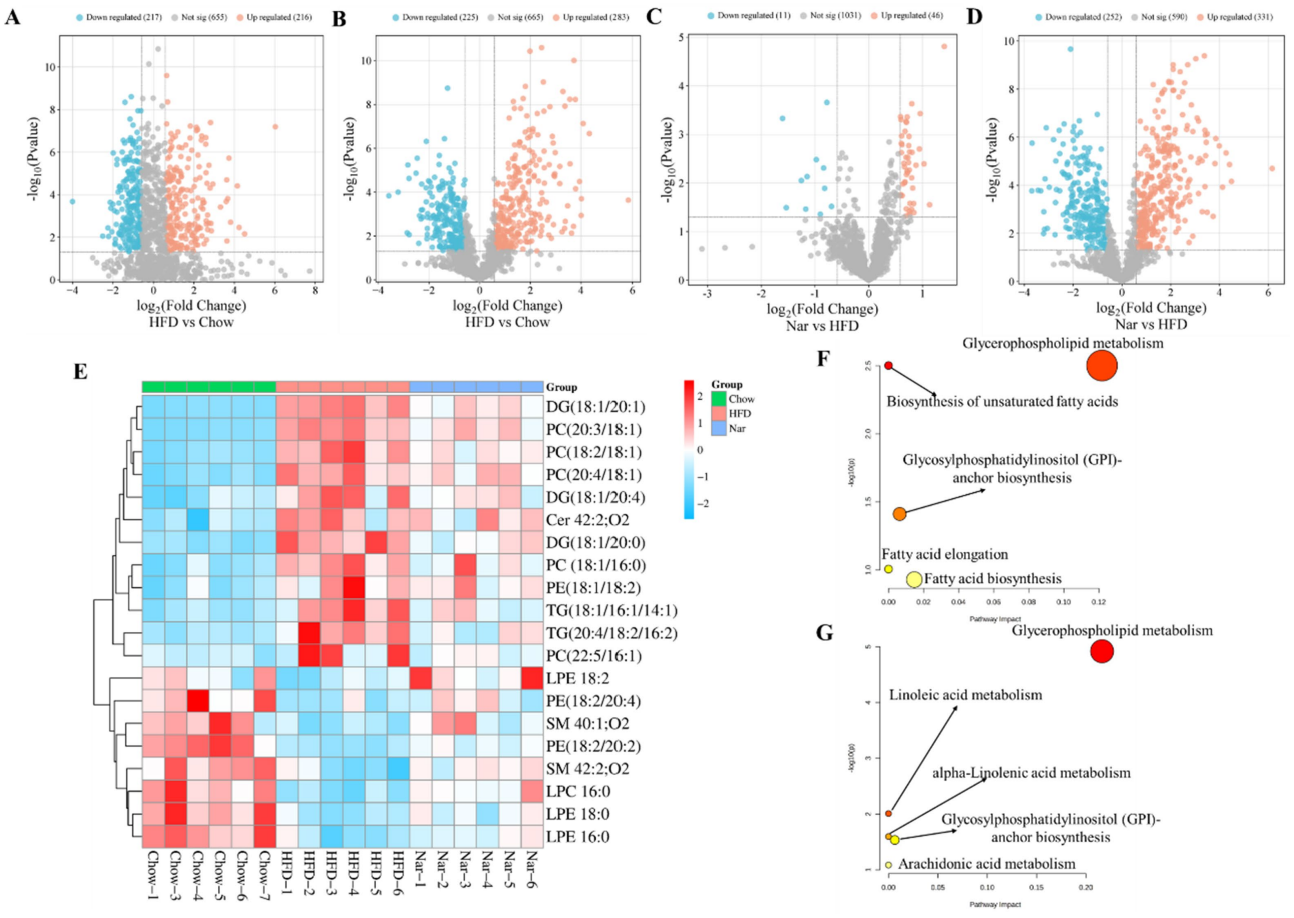
Comprehensive analysis of differential lipid profiles across the Chow, HFD, and Nar groups in both positive and negative ionization modes. **(A–D)** Volcano plots illustrating pairwise comparisons: **(A)** HFD vs. Chow (positive mode), **(B)** HFD vs. Chow (negative mode), **(C)** Nar vs. HFD (positive mode), and **(D)** Nar vs. HFD (negative mode). Significantly altered lipids (red) and non-significant lipids (blue) are shown. The horizontal dashed line corresponds to the significance threshold (−log₁₀(*p*-value)), and vertical dashed lines indicate the fold-change cutoffs. **(E)** Heatmap of hepatic lipid abundances across the experimental groups. **(F,G)** Pathway enrichment analysis of significantly altered lipid metabolic pathways between HFD vs. Chow **(F)** and Nar vs. HFD **(G)**. Functionally relevant pathways are annotated.

Orthogonal partial least squares discriminant analysis (OPLS-DA) revealed significant separation between the groups (Supplementary Figure S1), indicating distinct metabolic profiles across the Chow, HFD, and Nar groups. To identify potential lipid biomarkers, we selected lipid species based on Fold Change (FC) ≥ 1.5 or ≤0.67and *p* < 0.05. A total of 526 differential lipid species distinguished the Chow and HFD groups, while 283 lipid species differentiated the Nar and HFD groups (Figures 4A–D). Based on variable importance in projection (VIP) > 1 and FC ≥ 1.5 or ≤0.67, 20 lipid species were selected as potential biomarkers for Nar intervention (Table 1).

**Table 1:** Significantly changed hepatic lipid species regulated by the Nar.

NO.	TR	Adducts	Adducts	Adducts	HFD/Chow				Nar/HFD			
					VIP	FDR	FC	Trend	VIP	FDR	FC	Trend
1	13.38	[M + FA]^−^	Cer 42:2; O2	C_42_H_81_NO_3_	2.03	0.0017	1.53	↑	1.59	0.0013	0.45	↓
2	13.91	[M + NH4]^+^	DG (18:1/20:0)	C_41_H_78_O_5_	1.48	0.0000	5.14	↑	1.06	0.0043	0.62	↓
3	13.2	[M + NH4]^+^	DG (18:1/20:1)	C_41_H_76_O_5_	4.12	0.0000	9.78	↑	2.94	0.0002	0.64	↓
4	11.56	[M + NH4]^+^	DG (18:1/20:4)	C_41_H_70_O_5_	1.96	0.0014	1.83	↑	1.49	0.0628	0.80	↓
5	3.01	[M + H]^+^	LPC 16:0	C_24_H_50_NO_7_P	4.11	0.0002	0.67	↓	2.18	0.0016	1.53	↑
6	3.14	[M + H]^+^	LPE 16:0	C_21_H_44_NO_7_P	3.28	0.0002	0.56	↓	1.43	0.0264	1.20	↑
7	4.3	[M + H]^+^	LPE 18:0	C_23_H_48_NO_7_P	4.24	0.0004	0.50	↓	2.37	0.0327	1.31	↑
8	2.64	[M + H]^+^	LPE 18:2	C_23_H_44_NO_7_P	1.07	0.0477	0.85	↓	1.93	0.0167	1.31	↑
9	10.98	[M + H]^+^	PC (18:1/16:0)	C_42_H_82_NO_8_P	3.51	0.0000	1.66	↑	2.4	0.0374	0.85	↓
10	10.41	[M + H]^+^	PC (18:2/18:1)	C_44_H_82_NO_8_P	4.19	0.0000	2.21	↑	2.3	0.0051	0.89	↓
11	10.6	[M + H]^+^	PC (20:3/18:1)	C_46_H_84_NO_8_P	2.51	0.0000	3.64	↑	1.48	0.0150	0.79	↓
12	10.08	[M + H]^+^	PC (20:4/18:1)	C_46_H_82_NO_8_P	2.86	0.0000	2.61	↑	1.4	0.0485	0.91	↓
13	9.69	[M + FA]^−^	PC (22:5/16:1)	C_46_H_80_NO_8_P	1.82	0.0284	1.42	↑	3.7	0.0001	0.78	↓
14	10.61	[M + H]^+^	PE (18:1/18:2)	C_41_H_76_NO_8_P	1.89	0.0040	1.69	↑	1.18	0.0473	0.90	↓
15	11.58	[M-H]^−^	PE (18:2/20:2)	C_43_H_78_NO_8_P	2.19	0.0000	0.31	↓	2.83	0.0002	1.74	↑
16	10.14	[M + H]^+^	PE (18:2/20:4)	C_43_H_74_NO_8_P	1.96	0.0178	0.73	↓	1.05	0.0478	1.16	↑
17	12.97	[M + FA]^−^	SM 40:1; O2	C_45_H_91_N_2_O_6_P	1.26	0.0022	0.43	↓	1.79	0.0406	1.79	↑
18	12.81	[M + FA]-	SM 42:2; O2	C_47_H_93_N_2_O_6_P	1.74	0.0047	0.76	↓	1.46	0.0369	1.19	↑
19	15.05	[M + NH4]^+^	TG (18:1/16:1/14:1)	C_51_H_92_O_6_	2.42	0.0005	2.38	↑	2.01	0.0306	0.68	↓
20	14.83	[M + NH4]^+^	TG (20:4/18:2/16:2)	C_57_H_94_O_6_	1.87	0.0004	3.00	↑	1.74	0.0143	0.56	↓

A heatmap was generated to visualize changes in lipid classes in the liver of mice (Figure 4E). The results indicated that the levels of Cer 42:2; O2, LPE 16:0, LPE 18:0, LPE 18:2, PE (18:2/20:2), PE (18:2/20:4), LPC 16:0, SM 42:2; O2, and SM 40:1; O2 were significantly higher in the HFD group than the Chow group (*p* < 0.05). However, these lipid levels were significantly reduced following Nar supplementation (*p* < 0.05). Conversely, the levels of DG (18:1/20:0), DG (18:1/20:1), DG (18:1/20:4), PC (22:5/16:1), PC (18:1/16:0), PC (18:2/18:1), PC (20:3/18:1), PC (20:4/18:1), PE (18:1/18:2), TG (18:1/16:1/14:1), and TG (20:4/18:2/16:2) were significantly decreased in the HFD group compared to the Chow group but increased after Nar supplementation. These 20 lipid species, which include 4 glycolipids (GLs), 12 glycerophospholipids (GPs), and 4 sphingolipids (SPs), may serve as potential lipid biomarkers for Nar intervention. These findings suggest that Nar intervention effectively regulates hepatic lipid metabolism and ameliorates lipid metabolic abnormalities in MASLD mice. To identify changes in important pathways related to lipid metabolism, pathway enrichment analysis was performed.

Compared to the Chow group, the HFD group significantly impacted pathways involved in unsaturated fatty acid biosynthesis, glycerophospholipid metabolism, glycosylphosphatidylinositol (GPI)-anchor protein biosynthesis, fatty acid elongation, and fatty acid biosynthesis (Figures 4F,G). Nar supplementation alleviated many of these metabolic alterations, with glycerophospholipid metabolism emerging as a potential target pathway, based on impact value scores. Overall, these results indicated that glycerophospholipid metabolism may play a crucial role in MASLD progression and in the protective effects of Nar.

### 3.4 Nar altered the gut microbiota composition in MASLD mice

**Figure 5:**
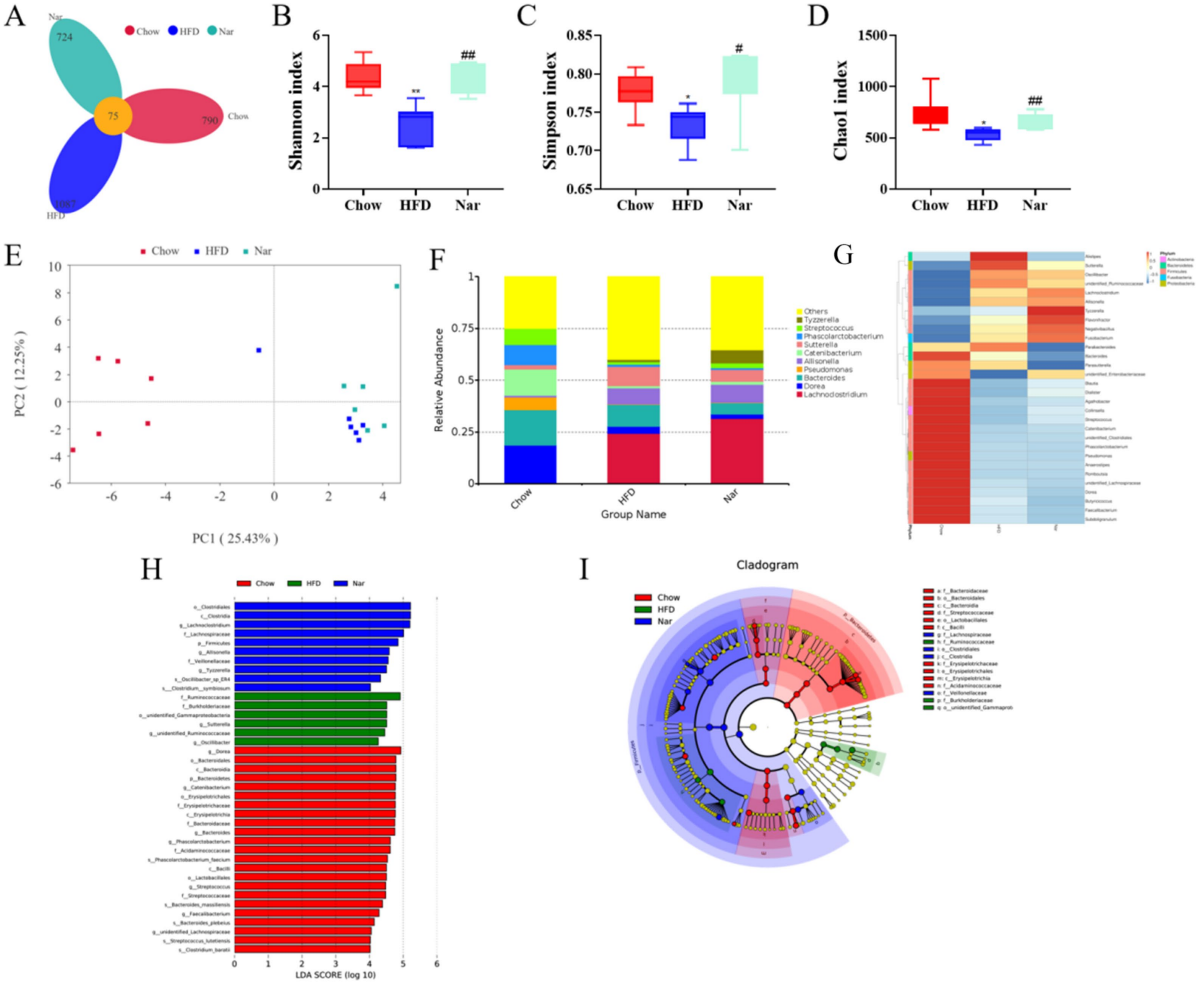
Nar increased gut microbiota diversity of MASLD mice. **(A)** Venn diagram illustrating the gut microbiota composition across different groups of mice. **(B)** Shannon index. **(C)** Simpson index. **(D)** Chao 1 index. **(E)** Unweighted UniFrac principal coordinate analysis (PCoA) of gut microbiota. **(F)** Analysis of the relative abundance of gut microbiota at the genus level. **(G)** Heatmap depicting the species composition of gut microbiota at the genus level. **(H)** Histogram of linear discriminant analysis (LDA) effect sizes for marked species. **(I)** LEfSe analysis. Statistical significance is indicated as follows: *p < 0.05, **p < 0.01 compared to the Chow group; ^#^*p* < 0.05, ^##^*p* < 0.01 compared to the HFD group.

Alterations in gut microbiota are closely associated with the development of MASLD (22). To explore the mechanism by which Nar alleviates MASLD, we analyzed the gut microbiota in mice using intestinal content samples. As shown in Figure 5A, a total of 2,601 bacterial operational taxonomic units (OTUs) were identified, with 865 OTUs in the Chow group and 1,162 OTUs in the HFD group. Among them, 75 OTUs were shared across all three groups (Figure 5A). To investigate the effect of Nar on α-diversity of gut microbiota in HFD mice, including the observed species index and the Chao1, Shannon, and Simpson indices, we found that the Nar group showed significant increases in these indices when compared to the HFD group (Figures 5B–D). Principal coordinates analysis (PCoA) showed that the gut microbiota compositions and structures of the three groups were significantly different (Figure 5E). At the genus level (Figure 5F), Nar supplementation increased the number of bacterial species compared to the Chow and HFD groups, suggesting that Nar may have a beneficial effect on gut microbiota composition. We further analyzed the abundance of the top 25 genera across the different groups, revealing significant differences between the HFD and Chow groups (Figure 5G). Notably, the microbiota producing short-chain fatty acids (SCFAs), including *Oscillibacter*, *Allisonell*, *unidentified Ruminococcaceae*, and *Allisonella,* increased in the Nar group compared to the HFD group, which indicated that gut microbiota dysbiosis had occurred in the HFD group. The taxonomic distribution of enriched and marker species is shown in Figures 5G,I. Linear discriminant analysis (LDA) with a threshold of >4 revealed significant differences (*p* < 0.05) in the abundance of *Lachnoclostridium*, *Allisonella*, and *Tyzzerella* in the Nar group; *Sutterella*, *Coriobacteriales*, an *unidentified Ruminococcaceae* genus, and *Oscillibacter* in the HFD group; and *Bacteroides* in the Chow group.

## 4 Discussion

Metabolic dysfunction-associated steatotic liver disease (MASLD) is a heterogeneous chronic condition closely associated with global metabolic abnormalities (23). Despite its high prevalence, effective pharmacological treatments remain elusive. While Nar has shown promise in ameliorating MASLD, its underlying mechanisms require further clarification (24). However, the precise mechanisms remain unclear. This study combines lipidomics, network pharmacology, and gut microbiomics to further investigate the therapeutic mechanisms of Nar in MASLD.

Our study indicated that Nar supplementation effectively reduces body weight, epididymal fat tissue weight, serum AST, and ALT levels while also improving hepatic steatosis by decreasing liver weight and hepatic TG levels. Metabolic disturbances and toxic lipid accumulation are considered central factors in the development of MASLD (25). Consequently, lipidomics is commonly used to link lipid dysregulation with the pathological changes in the MASLD liver. Through lipidomic analysis, we identified 22 distinct lipid species in the liver of mice, including TGs, diglycerides (DGs), phosphatidylcholines (PCs), phosphatidylethanolamines (PEs), sphingomyelins (SMs), and ceramides (Cer). These lipid species effectively differentiated Nar-treated mice from MASLD mice. Notably, the accumulation of TGs in the liver is regarded as the initial step in the progression of MASLD (26). Several studies have shown that TG levels are elevated in MASLD patients, and numerous studies have investigated the potential of using TGs as biomarkers for MASLD (27). In our study, the majority of TG species increased in the liver of HFD-induced MASLD mice. Our lipidomic data revealed that Nar supplementation significantly exhibited anti-dyslipidemic effects by reducing the levels of TG (18:1/16:1/14:1) and TG (20:4/18:2/16:2) in the liver of HFD-induced MASLD mice. Growing evidence suggests that elevated levels of monounsaturated DGs in the liver are associated with MASLD (28). Notably, our data indicated an increase in the levels of monounsaturated DGs (e.g., DG (18:1/20:0), DG (18:1/20:1), and DG (18:1/20:4)) in the liver of MASLD mice, suggesting that DGs may play a crucial role in the anti-MASLD effects of Nar.

Glycerophospholipids, such as PCs and PEs, are essential for maintaining the structure and function of cell membranes (29). Dysregulation of glycerophospholipid metabolism can impair the synthesis and secretion of very-low-density lipoproteins (VLDL), thereby promoting the progression of MASLD (30). Our findings indicate that the level of PC is reduced in the liver of MASLD mice. When PC levels are insufficient, adequate VLDL cannot be synthesized to transport hepatic TGs, leading to excessive lipid deposition in the liver. However, supplementation with Nar restored the levels of PCs and PEs, suggesting that Nar exerts its effects by modulating the dynamic balance between PCs and TGs in the liver.

Based on the “disease-gene-target-drug” network system, network pharmacology is widely used to comprehensively investigate the effects of drugs on diseases (31). Our study demonstrates that Nar, a bioactive flavonoid derived from citrus, may exert therapeutic effects on MASLD through a multi-target and multi-pathway mechanism. Integrative analysis of lipidomics and network pharmacology identified the PI3K-AKT signaling pathway as a key regulatory axis, which is essential for hepatic insulin regulation by mediating glucose transport, β-cell secretion, and insulin gene transcription (32). Molecular docking further validated the network pharmacology predictions, showing strong binding affinities of Nar with core targets such as PIK3CA, ESR1, MMP9, and SRC, without significant toxicity risks. Functionally, SRC acts upstream of the PI3K-AKT pathway via ATP-dependent phosphorylation of substrates (33), whereas MMP9 contributes to adipose tissue remodeling and fibrosis through extracellular matrix degradation, processes that are tightly linked to chronic inflammation, insulin resistance, and lipid metabolic disorders (34). Collectively, these findings suggest that Nar may ameliorate MASLD by modulating the PI3K-AKT signaling cascade through direct interactions with key proteins, providing novel mechanistic insights and potential therapeutic implications.

Gut microbiota dysbiosis has been increasingly recognized as a critical contributor to the pathogenesis of MASLD and other metabolic disorders (35). Our findings suggest that naringin may reach the colon in its intact form, where it is metabolized by specific bacterial species to exert prebiotic effects, which is consistent with previous reports (36). Nar supplementation significantly enhanced microbial diversity and enriched beneficial short-chain fatty acid (SCFA)-producing genera, including *Oscillibacter* and *Allisonella*. Previous studies have demonstrated that increased SCFA production strengthens the intestinal barrier and attenuates systemic inflammation, thereby alleviating MASLD progression (37). Consistent with these findings (38), we observed that Nar intervention led to marked alterations in gut microbial abundance, highlighting the pivotal role of the gut–liver axis in mediating its therapeutic efficacy against MASLD. Overall, this study suggests that Nar may help prevent obesity-related complications by maintaining gut microbiota homeostasis, enriching specific probiotic populations, balancing hepatic lipid profiles, and enhancing certain hepatic lipid classes in MASLD mice.

## 5 Conclusion

Our study demonstrates that naringin protects against HFD-induced MASLD by restoring hepatic lipid homeostasis and reshaping the gut microbiota. Lipidomic analysis identified 20 differentially abundant lipid species as potential biomarkers, while microbiome profiling revealed an enrichment of beneficial genera such as *Oscillibacter* and *Allisonella*. Molecular docking and bioinformatic analyses further indicated interactions of naringin with core targets, including PIK3CA, ESR1, MMP9, and CSR, suggesting plausible mechanistic pathways. Although the exploration of the relationship between Nar and “gut–liver axis” as a promising therapeutic target remains largely theoretical, these findings highlight naringin as a promising therapeutic candidate for MASLD and underscore the utility of integrating multi-omics with experimental pharmacology to advance mechanistic insights into complex metabolic diseases.

## Data Availability

The original contributions presented in the study are included in the article/Supplementary material, further inquiries can be directed to the corresponding author.

## References

[1] Eskridge W Cryer DR Schattenberg JM Gastaldelli A Malhi H Allen AM. Metabolic dysfunction-associated Steatotic liver disease and metabolic dysfunction-associated Steatohepatitis: the patient and physician perspective. J Clin Med. (2023) 12:6216. doi: 10.3390/jcm12196216, PMID: 37834859 PMC10573476

[2] Tan EY Muthiah MD Sanyal AJ. Metabolomics at the cutting edge of risk prediction of MASLD. Cell Rep Med. (2024) 5:101853. doi: 10.1016/j.xcrm.2024.101853, PMID: 39657668 PMC11722125

[3] Fan J-G Kim S-U Wong VW-S. New trends on obesity and NAFLD in Asia. J Hepatol. (2017) 67:862–73. doi: 10.1016/j.jhep.2017.06.003, PMID: 28642059

[4] Cotter TG Rinella M. Nonalcoholic fatty liver disease 2020: the state of the disease. Gastroenterology. (2020) 158:1851–64. doi: 10.1053/j.gastro.2020.01.052, PMID: 32061595

[5] Younossi ZM Golabi P de Avila L Paik JM Srishord M Fukui N. The global epidemiology of NAFLD and NASH in patients with type 2 diabetes: a systematic review and Meta-analysis. J Hepatol. (2019) 71:793–801. doi: 10.1016/j.jhep.2019.06.021, PMID: 31279902

[6] Ni Y Qian L Siliceo SL Long X Nychas E Liu Y. Resistant starch decreases intrahepatic triglycerides in patients with NAFLD via gut microbiome alterations. Cell Metab. (2023) 35:1530–1547.e8. doi: 10.1016/j.cmet.2023.08.002, PMID: 37673036

[7] Nakatsuka T Tateishi R Koike K. Changing clinical management of NAFLD in Asia. Liver Int. (2022) 42:1955–68. doi: 10.1111/liv.15046, PMID: 34459096

[8] Rong L Zou J Ran W Qi X Chen Y Cui H. Advancements in the treatment of non-alcoholic fatty liver disease (NAFLD). Front Endocrinol (Lausanne). (2022) 13:1087260. doi: 10.3389/fendo.2022.1087260, PMID: 36726464 PMC9884828

[9] Nobili V Alisi A Valenti L Miele L Feldstein AE Alkhouri N. NAFLD in children: new genes, new diagnostic modalities and new drugs. Nat Rev Gastroenterol Hepatol. (2019) 16:517–30. doi: 10.1038/s41575-019-0169-z, PMID: 31278377

[10] Nie Q Chen H Hu J Fan S Nie S. Dietary compounds and traditional Chinese medicine ameliorate type 2 diabetes by modulating gut microbiota. Crit Rev Food Sci Nutr. (2019) 59:848–63. doi: 10.1080/10408398.2018.1536646, PMID: 30569745

[11] Liebe R Esposito I Bock HH Vom Dahl S Stindt J Baumann U. Diagnosis and Management of Secondary Causes of Steatohepatitis. J Hepatol. (2021) 74:1455–71. doi: 10.1016/j.jhep.2021.01.045, PMID: 33577920

[12] Adak A Khan MR. An insight into gut microbiota and its functionalities. Cell Mol Life Sci. (2019) 76:473–93. doi: 10.1007/s00018-018-2943-4 30317530 PMC11105460

[13] Perler BK Friedman ES Wu GD. The role of the gut microbiota in the relationship between diet and human health. Annu Rev Physiol. (2023) 85:449–68. doi: 10.1146/annurev-physiol-031522-092054 36375468

[14] Yang Y Trevethan M Wang S Zhao L. Beneficial effects of Citrus flavanones Naringin and Naringenin and their food sources on lipid metabolism: an update on bioavailability, pharmacokinetics, and mechanisms. J Nutr Biochem. (2022) 104:108967. doi: 10.1016/j.jnutbio.2022.108967, PMID: 35189328 PMC9058202

[15] Pengnet S Sumarithum P Phongnu N Prommaouan S Kantip N Phoungpetchara I. Naringin attenuates fructose-induced NAFLD progression in rats through reducing endogenous triglyceride synthesis and activating the Nrf2/HO-1 pathway. Front Pharmacol. (2022) 13:1049818. doi: 10.3389/fphar.2022.1049818, PMID: 36588703 PMC9797507

[16] Lam SM Wang Z Li B Shui G. High-coverage Lipidomics for functional lipid and pathway analyses. Anal Chim Acta. (2021) 1147:199–210. doi: 10.1016/j.aca.2020.11.024, PMID: 33485579

[17] Liu H Chen T Xie X Wang X Luo Y Xu N. Hepatic Lipidomics analysis reveals the ameliorative effects of Highland barley β-Glucan on Western diet-induced nonalcoholic fatty liver disease mice. J Agric Food Chem. (2021) 69:9287–98. doi: 10.1021/acs.jafc.1c03379, PMID: 34347479

[18] Wang R Li B Lam SM Shui G. Integration of Lipidomics and metabolomics for in-depth understanding of cellular mechanism and disease progression. J Genet Genomics. (2020) 47:69–83. doi: 10.1016/j.jgg.2019.11.009, PMID: 32178981

[19] Jiang S Fan X Hua J Liu S Feng Y Shao D. Integrated metabolomics and network pharmacology analysis to reveal the protective effect of Complanatoside a on nonalcoholic fatty liver disease. Eur J Pharmacol. (2024) 985:177074. doi: 10.1016/j.ejphar.2024.177074, PMID: 39481627

[20] Mu H Zhou Q Yang R Zeng J Li X Zhang R. Naringin attenuates high fat diet induced non-alcoholic fatty liver disease and gut bacterial Dysbiosis in mice. Front Microbiol. (2020) 11:585066. doi: 10.3389/fmicb.2020.585066, PMID: 33281780 PMC7691324

[21] Deprince A Haas JT Staels B. Dysregulated lipid metabolism links NAFLD to cardiovascular disease. Mol Metab. (2020) 42:101092. doi: 10.1016/j.molmet.2020.101092, PMID: 33010471 PMC7600388

[22] Chen Y Zhou J Wang L. Role and mechanism of gut microbiota in human disease. Front Cell Infect Microbiol. (2021) 11:625913. doi: 10.3389/fcimb.2021.625913, PMID: 33816335 PMC8010197

[23] Guo X Yin X Liu Z Wang J. Non-alcoholic fatty liver disease (NAFLD) pathogenesis and natural products for prevention and treatment. Int J Mol Sci. (2022) 23:15489. doi: 10.3390/ijms232415489, PMID: 36555127 PMC9779435

[24] Guan L Guo L Zhang H Liu H Zhou W Zhai Y. Naringin protects against non-alcoholic fatty liver disease by promoting Autophagic flux and Lipophagy. Mol Nutr Food Res. (2024) 68:e2200812. doi: 10.1002/mnfr.202200812 38054638

[25] Jones JG. Hepatic glucose and lipid metabolism. Diabetologia. (2016) 59:1098–103. doi: 10.1007/s00125-016-3940-5, PMID: 27048250

[26] Zhang C Wang K Yang L Liu R Chu Y Qin X. Lipid metabolism in inflammation-related diseases. Analyst. (2018) 143:4526–36. doi: 10.1039/c8an01046c, PMID: 30128447

[27] Feng S Gan L Yang CS Liu AB Lu W Shao P. Effects of Stigmasterol and β-Sitosterol on nonalcoholic fatty liver disease in a mouse model: a Lipidomic analysis. J Agric Food Chem. (2018) 66:3417–25. doi: 10.1021/acs.jafc.7b06146, PMID: 29583004

[28] Li T Guo W Zhou Z. Adipose triglyceride lipase in hepatic physiology and pathophysiology. Biomolecules. (2021) 12:57. doi: 10.3390/biom12010057, PMID: 35053204 PMC8773762

[29] Tabe S Hikiji H Ariyoshi W Hashidate-Yoshida T Shindou H Shimizu T. Lysophosphatidylcholine acyltransferase 4 is involved in Chondrogenic differentiation of ATDC5 cells. Sci Rep. (2017) 7:16701. doi: 10.1038/s41598-017-16902-4, PMID: 29196633 PMC5711957

[30] London A Lundsgaard A-M Kiens B Bojsen-Møller KN. The role of hepatic fat accumulation in glucose and insulin homeostasis-dysregulation by the liver. J Clin Med. (2021) 10:390. doi: 10.3390/jcm10030390, PMID: 33498493 PMC7864173

[31] Zhang P Zhang D Zhou W Wang L Wang B Zhang T. Network pharmacology: towards the artificial intelligence-based precision traditional Chinese medicine. Brief Bioinform. (2023) 25:bbad518. doi: 10.1093/bib/bbad518 38197310 PMC10777171

[32] Li Y Li W Zhu X Xu N Meng Q Jiang W. VEGFB ameliorates insulin resistance in NAFLD via the PI3K/AKT signal pathway. J Transl Med. (2024) 22:976. doi: 10.1186/s12967-024-05621-w, PMID: 39468621 PMC11520811

[33] Han MW Ryu IS Lee JC Kim SH Chang HW Lee YS. Phosphorylation of PI3K regulatory subunit P85 contributes to resistance against PI3K inhibitors in radioresistant head and neck cancer. Oral Oncol. (2018) 78:56–63. doi: 10.1016/j.oraloncology.2018.01.014, PMID: 29496059

[34] Rodrigues-Junior DM Tsirigoti C Psatha K Kletsas D Aivaliotis M Heldin C-H. TGF-β induces cholesterol accumulation to regulate the secretion of tumor-derived extracellular vesicles. J Exp Clin Cancer Res. (2025) 44:42. doi: 10.1186/s13046-025-03291-0, PMID: 39910665 PMC11800471

[35] Huang F Zheng X Ma X Jiang R Zhou W Zhou S. Theabrownin from Pu-Erh tea attenuates hypercholesterolemia via modulation of gut microbiota and bile acid metabolism. Nat Commun. (2019) 10:4971. doi: 10.1038/s41467-019-12896-x, PMID: 31672964 PMC6823360

[36] Marín L Miguélez EM Villar CJ Lombó F. Bioavailability of dietary polyphenols and gut microbiota metabolism: antimicrobial properties. Biomed Res Int. (2015) 2015:905215. doi: 10.1155/2015/905215, PMID: 25802870 PMC4352739

[37] Wang G Yu Y Wang Y-Z Wang J-J Guan R Sun Y. Role of SCFAs in gut microbiome and glycolysis for colorectal Cancer therapy. J Cell Physiol. (2019) 234:17023–49. doi: 10.1002/jcp.28436, PMID: 30888065

[38] Loo YT Howell K Chan M Zhang P Ng K. Modulation of the human gut microbiota by Phenolics and phenolic Fiber-rich foods. Compr Rev Food Sci Food Saf. (2020) 19:1268–98. doi: 10.1111/1541-4337.12563, PMID: 33337077

