# Supplementary material for: Integrated multi-omics analysis reveals the mechanisms of naringin in ameliorating high-fat diet-induced metabolic dysfunction-associated steatotic liver disease
Source: Front Nutr. 2025 Oct 21;12:1694191. doi: 10.3389/fnut.2025.1694191 (PMC12583168; doi:10.3389/fnut.2025.1694191)
Supplement: Supplementary file 1 [file Table_1.DOC]

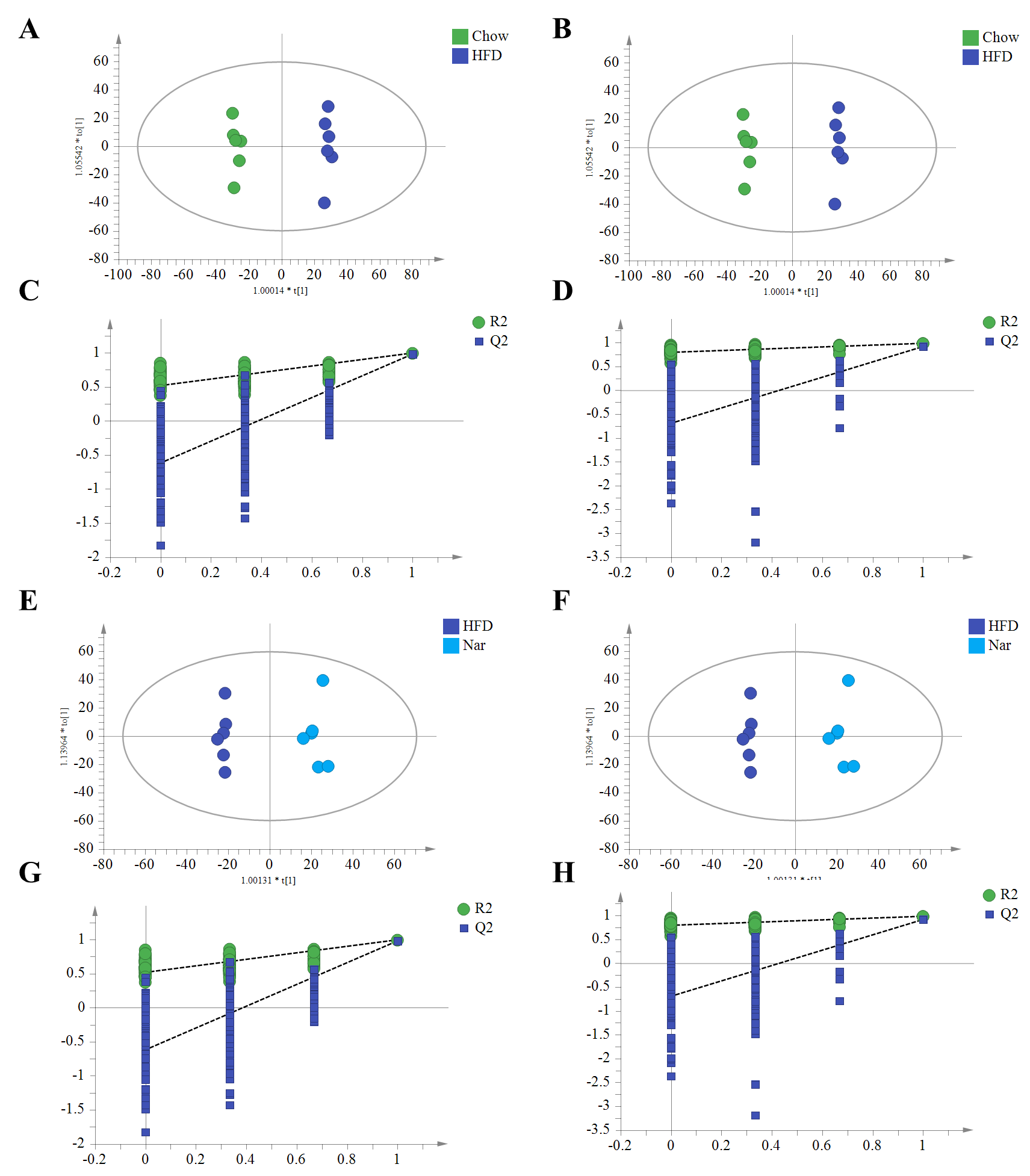


Fig S1. Score plots of the OPLS-DA models of the three groups; (A) OPLS-DA score plot between HFD group and Chow group in positive ion mode. (B) OPLS-DA score plot between HFD group and Chow group in negative ion mode. (C) 200 permutations of liver lipids OPLS-DA model between HFD group and Chow group in positive ion mode. (D) 200 permutations of liver lipids OPLS-DA model between HFD group and Chow group in negative ion mode. (E) OPLS-DA score plot between Nar group and HFD group in positive ion mode. (F) OPLS-DA score plot between Nar group and HFD group in negative ion mode. (G) 200 permutations of liver lipids OPLS-DA model between Nar group and HFD group in positive ion mode. (H) 200 permutations of liver lipids OPLS-DA model between Nar group and HFD group in negative ion mode.

**Table S1. Primer Sequences**

| **Primer Name** | **Forward** | **Revers** |
| --- | --- | --- |
| *ACCα* | 5’-TATCAGTTTCCCAGCCAGCA-3’ | 5’-ATCCTACACCACAGCCTTCA-3’ |
| *FAS* | 5'-CACAGCAACCAGCAATACAAA-3' | 5'-TTCAGCAATTCTCGGGATGT-3' |
| *PPARα* | 5’-GCATGTGAAGGCTGTAAGGG-3’ | 5’-TTGTGTGACATCCCGACAGA-3’ |
| *CPT-1α* | 5’-AGTCCTGCAACTTTGTGCTG-3’ | 5’-GGTGAGTCGACTGCCAGATA-3’ |
